# Effects of Different Powder to liquid Ratios on the Push out Bond Strength of CEM Cement on Simulated Perforations in the Furcal Area

**DOI:** 10.4317/jced.53805

**Published:** 2017-06-01

**Authors:** Mohammad-Frough Reyhani, Negin Ghasemi, Vahid Zand, Saba Mosavizadeh

**Affiliations:** 1 Associate Professor, Department of Endodontics, Dental Faculty, Tabriz University (Medical Sciences), Tabriz, Iran; 2 Assistant Professor, Department of Endodontics, Dental and Periodontal Research Center, Dental Faculty, Tabriz University (Medical Sciences), Tabriz, Iran; 3 Associate Professor, Department of Endodontics, Dental Faculty, Tabriz University (Medical Sciences), Tabriz, Iran; 4 Private practice, Tabriz, Iran

## Abstract

**Background:**

Proper bond strength to dentin is one of the properties of biomaterials used for therapeutic purposes such as repair of furcal perforations. The aim of the present study to evaluate the effects of different powder to liquid ratios of Calcium-enriched mixture (CEM) on the push-out bond strengths in simulated perforations in the furcal area and compare it with Mineral trioxide aggregate (MTA).

**Material and Methods:**

Furcal perforations, measuring 1.3 mm in diameter and 2 mm in height, were prepared in 120 mandibular first molars. Then the samples were randomly assigned to two groups (n=60). MTA and CEM cement were used for the repair of perforations in groups 1 and 2, respectively. Each group was divided into 3 subgroups based on the powder to liquid rations as follows: subgroup 1 (W/P) , 1:2; subgroup 2, 1:3; and subgroup 3, 1:4. After mixing and placing the materials in the perforation area, the samples were placed in closed containers at 37°C and 100% relative humidity for one week. A universal testing machine was used to determine the bond strength values. After recording the standard deviations, data were analyzed with two-way ANOVA. Statistical significance was set at *P*<0.05.

**Results:**

In the MTA and CEM groups the highest push-out bond strength values were recorded in the third (W/P: 1:4) and first (W/P: 1:2) subgroups, respectively. There were significant differences in both groups between the ratios mentioned above and the other ratios (*P*<0.05).

**Conclusions:**

Under the limitations of the present study, an increase in the powder-to-liquid ratio in CEM cement resulted in a decrease in bond strength, contrary to MTA.

** Key words:**CEM, Perforation, Powder-to-liquid ratio, push-out bond strength, MTA.

## Introduction

Proper bond strength to dentin is one of the properties of biomaterials used for therapeutic purposes such as repair of furcal perforations, pulp capping and apexogenesis of immature teeth because a favorable bonding capacity results in resistance to dislodgement under functional loads and the forces of placement of restorative materials, indirectly affecting the seal (1,2). One of the techniques used to determine the bond strength is the push-out test which is a reliable technique in this respect based on the results of previous studies (3).

Calcium-enriched Mixture (CEM) and Mineral Trioxide Aggregate (MTA) are two cements with different compositions of calcium, with similar applications in the field of root canal therapy (4,5). Their application for the repair of perforations and root canal therapy of mature teeth has yielded favorable treatment out comes (2,6). These two cements are hydrophilic and their powder particles set after being mixed with water. Therefore, the physical properties of the resultant mix are affected by the powder-to-liquid ratio (7,8). The manufacture has recommended a powder-to-liquid ratio of 3:1 for MTA. No specific ratio has been reported in relation to CEM.

In the majority of cases, the dentists’ favorable consistency draws attention in clinical procedures, rather than the manufacturer’s instructions (7). Therefore, some of studies have evaluated the effect of different powder-to-liquid rations on the physicochemical properties of MTA. Based on the results of these studies, an increase in the powder-to-liquid ratio of MTA results in an increase in radiopacity, antibacterial properties and compressive strength, and a decrease in setting time and porosity (7-10). On the other hand, the pH and calcium ion release decrease, too (7-10). There is no published data available on the effect of powder-to-liquid ratio of CEM cement.

In relation to MTA, a study evaluated the effects of different powder-to-liquid ratios on its push-out bond strength; the results showed an increase in resistance to dislodgment with an increase in the amount of powder (10). No such study is available on CEM. Therefore, the aim of the present study was to evaluate and compare the effects of different powder-to-liquid ratios of CEM and MTA on the push-out bond strengths in simulated perforations of furcal area, as two most commonly used biomaterials in endodontics.

## Material and Methods

-Selection of teeth and preparation of samples

A total of 120 mandibular first molars were selected based on inclusion criteria, consisting of the absence of root fusions, morphological or size anomalies, carious lesions in the furcation area and previous root canal treatment. After elimination of soft tissues, the teeth were immersed in 0.5% chloramine-T solution. The crowns of the teeth were removed with a diamond disk (SP 1600 Microtome, Leica, Nu Block, Germany) at cement-enamel junction (CEJ). The teeth were subsequently mounted in acrylic resin molds, with a 3-mm area at furcal area out of the acrylic resin in order to create a small space for a gelatin sponge (Geltamp, Roeko-Coltène/Whaledent, Langenau, Germany) that would be used as a matrix for packing the materials used for the repair of perforations. A #1/2 round bur was placed perpendicular to the furcation floor and parallel to the long axis of the tooth to produce perforations. Then #1, #2, #3 and #4 Peeso Reamers (Dentsply Maillefer, Ballaigues, Switzerland) were used to enlarge the perforations to achieve a diameter of 1.3 mm. The heights of the walls of perforations were measured with the use of a periodontal probe to standardize them at 2 mm. Samples measuring <2 mm in the height of dentinal wall were excluded from the study. If the thickness of the dentin was >2 mm, the extra dentin was eliminated with a disk. All the samples were rinsed with normal saline solution to remove the resultant debris.

-Mixing of the materials and repair of perforation

The samples were randomly divided into two groups (n=60) in terms of the material used for the repair of the perforated area. MTA (Angelus, Londrina, Paraná, Brazil) and CEM (Yektazist Dandan, Tehran, Iran) were used in groups 1 and 2, respectively. Each group was divided into 3 subgroups in terms of the powder-to-liquid ratio as follows.

Subgroup 1: W/P: 1:2

Subgroup 2: W/P: 1:3

Subgroup 3: W/P: 1:4

The powder was weighed with the use of a digital weighing machine and the amount of liquid was determined with a micropipette. The mixing procedures in all the samples were carried out manually by one operator in 15 seconds. A gelatinous sponge was placed beneath the perforated area. Then the materials were carried out to the perforation area with an MTA carrier and condensed in the area with the use of a condenser with a suitable size. After removing the excess material from the perforated area, a piece of cotton pellet impregnated with normal saline solution was placed beneath and over the perforated area. Then the samples were stored in closed containers at 37°C and 100% relative humidity for one week within an incubator.

-Push-out test 

A universal testing machine (Model H5K-S; Hounsfield Test Equipment, Surrey, England) was used for the push-out test. The restorative materials placed in the perforated area were subjected to a force at a crosshead speed of 0.5 mm/min in the apical direction parallel to the tooth long axis by using a cylindrical bar that measured 1.1 mm in diameter, until dislodgment was observed. The maximum force just before dislodgment was recorded in Newtons (N). The push-out bond strength was calculated in MPa using the following formula.

Bond strength (MPa)=force necessary for dislodgment (N)/bonded surface area (mm2), where bonded surface area was calculated as the d×h, where d is the diameter of the perforated area and h is the height of perforation site.

-Statistical analysis

The mean ± standard deviation of push-out bond strength of the study groups was calculated. Kolmogorov-Smirnov revealed normal distribution of data; therefore, two-way ANOVA was used to evaluate the significance of the effect of W/P on the push-out bond strength of the two biomaterials and to compare the subgroups. Statistical significance was set at *P*<0.05.

## Results

 Table 1 presents the mean and standard deviations of bond strength values. In the MTA group, the bond strength in the subgroup with a W/P ratio of 1:4 was significantly higher than that in the other subgroups; however, the two other subgroups did not exhibit any significant differences. In the CEM group, the highest resistance to dislodgment was seen in the subgroup with a W/P ratio of 1:2, which was significantly higher than those in the two other subgroups; there was no significant difference between the two other subgroups.

**Table 1:**
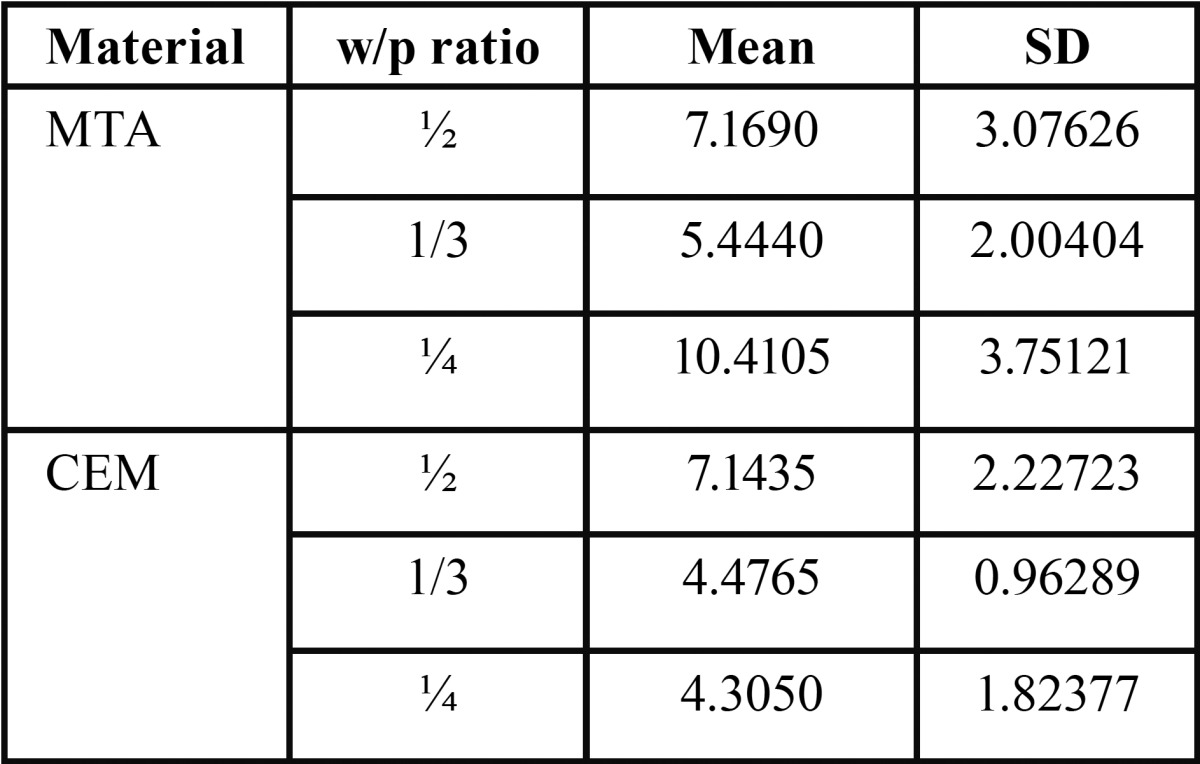
Means ± SD of the bond strength in study groups.

## Discussion

The present study was designed to evaluate the effect of different powder-to-liquid ratios on the push-out bond strength of MTA and CEM biomaterials in the repair of simulated perforations in the furcal area. The results showed an increase in resistance to displacement of MTA with an increase in the powder-to-liquid ratio; however, in CEM the lowest powder-to-liquid ratio evaluated resulted in the highest push-out bond strength.

Treatment of furcal area perforations has always been considered a challenge due to the risk of bone loss and destruction of periodontal tissues. One of the factors affecting the prognosis of treatment is the type of material used for the repair of the perforation (1,6). Such a material should be biocompatible and have the potential to induce osteogenesis and cementogenesis, in addition to the ability to bond to the cementum in the area and provide a proper seal (11). One of the techniques used to evaluate the bond strength is the push-out test (12,13) which was used in the present study. MTA and CEM cements are commonly used for the repair of perforations in the furcal area due to their proper physicochemical properties (1,14).

Since the two materials used in the present study are hydrophilic materials, it is logical that the powder-to-liquid ratio will affect their properties. The consistency of the cement is usually an operator-dependent property and the manufacturer’s instructions are ignored. Several studies have evaluated the effects of different powder-to-liquid ratios on the properties of MTA (7-10); However, no published data is available on CEM in this respect.

In the present study, CEM and MTA biomaterials we used for the repair of the perforated area. MTA is one of the most commonly used materials in endodontics (15). On the other hand, previous studies on the properties of CEM have reported that many of its properties are comparable to those of MTA (16,17). In the present study, the furcal areas in molar teeth were perforated, which is one of the most challenging and most common errors in endodontics (6).

Based on previous studies, some confounding factors affect the results of push-out test (3). One of these factors is the thickness of disks evaluated. In the present study the height of the perforated area was 2 mm because in clinical situations, the thickness of the furcal area is 2 mm in most cases, despite the fact that the recommended thicknesses is 1 mm because an increase in the thickness increases the risk of overestimation of the bond strength due to an increase in frictional forces. The diameter of the perforated area was standardized at 1.3 mm and the diameter of the Instron rod was 98% of this value, i.e. 1.1 mm to bring about the least confounding effect. The Instron rod exerted a force at a right angle to the furcal area floor with no interference with the walls.

The temperature and storage environment moisture of the samples were standardized. The materials were mixed manually in 15 second by one operator (18).

In the present study, an increase in the amount of powder resulted in an increase in the bond strength of MTA, consistent with the results of a study by Turker *et al.* (10). The ratios selected in the two studies were the same. An increase in the amount of water in MTA results in a decrease in cohesive strength between the particles, with a negative effect on the bond strength. In addition, an increase in water content increases the materials porosity, weakening the cement. One of the limitations of the present study and the study mentioned above was the absence of phosphate ions in the area. Under real clinical conditions the phosphate in the interstitial fluid comes into contact with MTA placed on the perorated area and results in an increase in biomineralization and bond strength of MTA (13,19). It is recommended that phosphate ions be placed in the area by placing a piece of cotton pellet impregnated with phosphate-buffered saline solution, rather than impregnated with normal saline solution, under the perforated area to better simulate the real clinical conditions and simulate the presence of phosphate ions (20,21).

In the present study, contrary to MTA, an increase in the amount of CEM powder resulted in a decrease in bond strength. One possible reason might be a difference in the chemical compositions of these two materials. Contrary to MTA, CEM contains phosphate ions in its chemical structures and can produce a hydroxyapatite layer in the absence of the interstitial fluid phosphate (1). An increase in the water ratio will result in the availability of a large amount of calcium hydroxide ions, which gives rise to the formation of hydroxyapatite in the presence of phosphate ions. There is no published data on CEM and it is not possible to compare the results.

The amount of water used for mixing should be balanced by considering the bioactivity, handling and other physical properties. It is important to achieve strong cement for the repair of perforated area. On the other hand, the material should resist displacement and provide a proper seal. Another important factor for success is the placement of a suitable coronal restoration after repairing the perforated area. The force used for condensing amalgam, by considering the size of the condenser is 5.5-9.17 MPa (1).

In the present study, MTA and CEM mixed at powder-to-liquid ratios of 4:1 and 2:1, receptively, achieved such strength. It is recommended that when these two materials are selected for the repair of perforations the ratios above be considered, rather than only use the 3:1 ratio recommended by the manufacturer.
